# A systems approach to the views of Jean-Jacques Rousseau on human-nature connection

**DOI:** 10.1007/s40656-026-00758-x

**Published:** 2026-09-07

**Authors:** Irina Salmi

**Affiliations:** 1 Department of Psychology and Speech-Language Pathology, University of Turku, Turku, Finland; 2 Turku Environmental Ethics Research Center, University of Turku, Turku, Finland

**Keywords:** Jean-Jacques Rousseau, The life-sustenance hypothesis, Human-nature connection, Biophilia, Altruism, Moral psychology

## Abstract

In environmental sciences, research on psychological and physical human-nature connection has recently expanded, revealing a diverse phenomenon associated with well-being, prosociality, and pro-environmentality. This paper argues that in his time, Jean-Jacques Rousseau (1712–1778) described a similar phenomenon with similar consequences, explaining its origin and importance in a nuanced manner that is able to bring new insights to the current theoretical frameworks on human-nature connection. In this paper, an interpretation of Rousseau’s philosophy with a systems approach is presented. Furthermore, by integrating it with temporary philosophy of systems biology and other relevant underpinnings, a framework for nature connectedness called the *life-sustenance hypothesis* is advanced, suggesting that all organisms are guided by two fundamental, parallel orientations: A self-sustaining orientation for self-preservative purposes and a life-sustaining orientation for sustaining surrounding life. Rousseau did not speak in these terms, but referred to different ‘manners-of-being’, the dynamics of which can be described with his central terms, *amour-de-soi*,* amour-propre*, and *pitié*. He suggested that the modern, individualistic lifestyle has introduced an excessive imbalance in these manners-of-beings, with harmful consequences on our well-being and the way we treat other beings. One possible solution Rousseau explored to resolve this imbalance—along with the political, educational, and domestic ones—was restoring personal inner peace, ‘harmony with Nature’. The experience of harmony, I suggest, greatly resembles the immersive psychological nature connectedness depicted by contemporary research. Rousseau’s system of thought is able to bring clarity into why we experience nature connectedness, and why it affects us the way it does.

## Introduction

Human-nature connection and its versatile effects on us and our behavior have been under cumulative multidisciplinary interest. Evidence regarding its beneficial effects is impressive: Associations have been found with physical and psychological well-being, prosocial behavior, and pro-environmental inclinations (see, e.g., a review of meta-analyses by Barragan-Jason et al., 2023). Research can be divided into studying (1) physical nature contact/exposure, and/or (2) a psychological experience of connectedness with nature. These are intertwined but separate phenomena that also influence us independently. Exposure enhances the experience of connectedness, and connectedness enhances the effects of exposure. In this paper, “human-nature connection” refers to both combined. Psychological nature connectedness is suggested to be a multidimensional (e.g., emotional, cognitive, experiential) phenomenon, studied with different methods related to different concepts that mostly correlate with one another—thus, an underlying, shared basis for them has been suggested (Tam, 2013). Furthermore, nature connectedness is described in different temporal terms: As a state of mind of immersive oneness with the natural world, or, something more stable, resembling personality traits (Whitburn et al., 2019). Theoretical attempts to comprehensively explain the phenomenon with all its varieties have not gained as much contemporary interest as the research itself: The aim of this paper is to address this gap.

In philosophy, one of the most distinguished advocates of human-nature connection is Jean-Jacques Rousseau (1712–1778). Rousseau, however, is not commonly mentioned in human-nature connection research, although connections have been drawn to modern environmental thinkers such as the deep ecologists (Lane & Clark, 2006; LaFreniere, 1990), who are referred to more often. I aim to offer insights into why Rousseau might be worth considering when attempting to understand the results of contemporary research.

My approach to Rousseau depicts him as a representative of the Western tradition of systems thinkers. While in the East, systems thinking has been more prevalent, currents also exist throughout Western philosophy and science, from Heraclitus and Spinoza all the way to contemporary scientific fields. Fritjof Capra and Pier Luigi Luisi (2014) have suggested that there is an ongoing paradigm shift towards a systems view of life, developing in different pace in different fields of science. They describe it in the following manner: “*The basic tension is one between the parts and the whole. The emphasis on the parts has been called mechanistic*,* reductionist*,* or atomistic; the emphasis on the whole*,* holistic*,* organismic*,* or ecological*.” They continue that systems thinking can be contrasted also with an individualistic approach. I suggest systems thinking is present in Rousseau’s philosophy in all these terms. In Rousseau’s (1979, p. 192) words, “*He who sees well the order of the whole sees the place where each part ought to be. He who sees a part well and knows it in depth may be a learned man; the other is a judicious man”*.

This paper is part of a project to develop a framework—the life-sustenance hypothesis—to explain human-nature connection and its versatile effects on us (Salmi, 2025a). The existing, most cited frameworks (such as the biophilia hypothesis, Stress Reduction Theory, Attention Restoration Theory, or Deep Ecology, introduced in the next section) all offer valuable insights but have not sufficiently addressed the multidimensionality, multitemporality, and multieffectiveness (well-being, prosociality, proenvironmentalism) of the phenomenon. The life-sustenance hypothesis was developed to answer these challenges. The main components of the hypothesis are Rousseau’s philosophy, Erich Fromm’s (1964) notion of biophilia, the philosophy of systems biology, and contemporary stress research. This paper will present the basis of the hypothesis that arises from Rousseau´s philosophy—further developed to meet the relevant, contemporary research and theoretical underpinnings—by taking an explanatory frame, based on his idea of “different manners-of-being”, to understand the relations between his key concepts, such as *amour-propre*,* amour-de-soi*,* pitié*, harmony, and true happiness.

Understanding the basic idea of the life-sustenance hypothesis will give structure to the proceeding of the paper.^1^ The systems biological basis of the hypothesis suggests that life is structured as interconnected nested systems within systems: All living entities (1) are constituted of parts and (2) are parts of larger wholes. This dual relation with the world—a whole with respect to its parts, a part with respect to the whole—expresses itself in all organisms as the dynamical tension between tendencies toward (1) biological *autonomy* and (2) *integration*. (Capra & Luisi, 2014; Boogerd et al., 2007; Moreno & Mossio, 2015.)^2^ According to the life-sustenance hypothesis, these tendencies induce adjacent orientations: the *self-sustaining* and the *life-sustaining* ones, guiding organisms simultaneously and species-specifically toward (1) self-preservation and (2) the sustenance of life beyond one’s own. *The experience of nature connectedness and altruistic impulses are suggested to be human manifestations of life-sustenance*. Moreover, relying on contemporary stress research (Sapolsky, 2004), many modern, individualistic cultures are suggested to contribute to an imbalance between these orientations.

Although not speaking in biological terms (biology as a discipline did not yet exist), Rousseau anticipated the tensions that current philosophy of systems biology emphasizes. Several scholars before me have drawn attention to these tensions in Rousseau, although not specifically naming him as a representative of systems thinking. For instance, Alexander Schmidt (2016, 3.1) discusses how Immanuel Kant was the first to depict as central in Rousseau a tension between humans as (1) individuals with individual needs, and at the same time, (2) members of our species, with aims beyond individual striving. This dilemma involves all living beings; it is inherent to life—we are autonomous organisms with self-sustaining goals, but we are also inevitably parts of our species, the ecosystems, and eventually, planetary life.

To introduce the reader to the systems approach to understanding Rousseau that subsequently led to the development of the life-sustenance hypothesis, I emphasize his unfinished book project called ‘*Morale Sensitive’ ou le Materialisme du Sage*, mentioned in his autobiographical *Confessions*. Through this choice, I want to show how Rousseau may have had ideas similar to the systems biological tendencies, although he, of course, used the language of his time to depict them. My focus is not on Rousseau’s political philosophy, but—in Judith Shklar’s (1985, p. 37) words—on his ‘moral psychology’: The innate drives, *amour-de-soi* (self-love in a self-preservative sense) and *pitié* (translated often as ‘compassion’). According to Rousseau, an excessive individualism (*amour-propre*) of modern times has caused severe problems, to which he explored several possible solutions to: Commonly acknowledged are the political and the educational solutions, but Shklar (1985, p. 63) suggests a third option: The return to the ‘Golden Age’ of small independent households represented in *Julie*,* or the New Heloise.* David Gauthier (2016, pp. 175–179) has emphasized yet another, fourth strategy that Rousseau explored, especially towards the end of his life: Withdrawing from society and focusing on finding inner peace (in the *Reveries of a Solitary*). Shklar (1985, p. 26) describes that this self-healing can recover one’s “true self”. Based on Rousseau’s *Moral Letters*, I suggest that this fourth path does not need the withdrawal to solitude to be permanent: Rather, it may offer a permanent change in one’s *mindset*. It is here that Rousseau takes up the issue of nature connectedness as a state of mind—*‘harmony’*—being a possible remedy for the ills of modern excessive individualism. In what follows, I will mainly concentrate on this fourth path, but will shortly reflect on the political writings in the Discussion. However, a detailed analysis of his political thinking in relation to the interpretative approach of this paper is left to future endeavours (for the importance of the endeavour, see Birler, 2025).

Before moving on, it is due time to address the definition of “nature” in this context. Rousseau used the word “nature” in several different meanings (Lähde, 2008). The aforementioned human-nature connection frameworks, however, refer more to “life” than “nature”. Systems approach defines life as a net-of-coexistence: Accordingly, “nature” might refer to the totality of this nested organization, or to the biosphere, entailing both biotic and abiotic components. Context-dependently, in this paper, nature refers to: (1) life as the parts of the biosphere that a human-organism is in direct contact with in situ (but, as it is in our cognitive nature—pun intended!—to anticipate what affects us, also indirect contact is included in “nature exposure”), or (2) the whole net-of-coexistence of which we are being part of and, thus, feel connected with.

In what follows, I will first provide a short introduction to the most often cited theoretical frameworks for human-nature connection with notifications on their shortcomings. Then, I will outline the broad interpretative angle of this paper to Rousseau’s works: the dual relationship with the world, inherent also in the philosophy of systems biology. Subsequently, Rousseau’s central concepts are introduced through the lens of this angle. In the Discussion, I come back to the potentiality of Rousseau’s system in explaining the results of contemporary human-nature connection studies.

## Earlier theoretical frameworks concerning human-nature connection

The idea of nature connectedness is often taken to be originated by Aldo Leopold (1887–1946) to whom most central in human existence is the tendency toward preserving or sustaining life (Leopold, 1949). Most often, however, the research refers to biologist E.O. Wilson’s (1984) biophilia hypothesis, where biophilia is defined as an evolutionary adaptive, innate need to affiliate with nature. Satisfying this need results in well-being. Biophilia connects to the savanna hypothesis (Ulrich, 1993), according to which we affiliate particularly with natural elements that remind us of the surroundings our ancestors relied on. Frequently cited theories are also the stress reduction theory, arguing that nature exposure improves well-being through decreasing stress responses (Ulrich et al., 1991), and attention restoration theory, according to which by reducing attentional fatigue, nature exposure leads to improved cognitive functioning and positive affect (Kaplan, 1995).

The theoretical basis for the association between pro-environmentalism and the human-nature connection is often drawn from the deep ecology movement initiated by Arne Næss (1973), who contrasts deep ecology with what he calls shallow ecology. Næss rejects atomistic individualism: Human beings are not radically separate from the rest of the world. While atomistic individualism leads to narrow, excessive selfishness, overcoming it leads to an *Ecological Self*, where nature becomes part of an individual’s self-identity. Næss proposed a relational total-field image of the world as a biospherical, co-existing net of knot-like organisms. Nature connectedness can be interpreted as a deep realization of this coexistence.

These most cited frameworks have been subject to criticism, and updates have been called for (e.g., Diehm, 2020). For instance, Wilson’s biophilia has been criticized for conceptual sloppiness and lack of proof in evolutionary adaptiveness terms (f.ex. Joye & DeBlock, 2011), and deep ecology has been confronted with problems in how to understand the Ecological Self: Is it, in the end, a self-centered approach after all, or does an individual point of view vanish altogether—both stances have been taken as problematic (see Diehm, 2020). Finally, most of the existing theoretical frameworks do not address in detail the variety of the human-nature connection revealed by contemporary research: I aim to show that Rousseau’s system of thought is able to bring clarity to the multidimensionality (e.g., cognitive, emotional, experiential), multitemporality (state-like/trait-like feature), and multieffectiveness (well-being, prosociality, proenvironmentalism). In the Discussion, we will come back to these questions.

## The dual relation with the world as a starting point

In my interpretation, at the center of Rousseau’s view of understanding life is *preservation*. All beings contribute to the survival of the whole: *“I see nothing which is not ordered according to the same system and does not contribute to the same end*—*namely*,* the preservation of the whole in its established order*” (Rousseau, 1979, p. 277). Dent (1988, p. 75) finds a similar preservation-emphasis in Rousseau’s use of the term ‘natural’: “*what is ‘natural’ is that which tends to the preservation*,* growth*,* amplitude*,* of secure*,* rich*,* being and life for a creature. What is unnatural defeats or destroys such a possibility*”.^3^ The centrality of preservation—not just in terms of self-sustaining, but also the preservation of the whole, i.e., general life-sustaining—leads to the interpretative lens of this paper: It offers a possible insight into why according to Rousseau (2012, pp. 474–475) the most useful work he could ever have offered to the world, would have been the unfinished *Morale Sensitive*, described in his *Confessions* (2012, pp. 474–475).^4^ Shklar (1985, p. 37) has pointed out that its message is also conveyed in *Emilé*, *Julie*, and the *Moral Letters*. I have used his other writings (along with the afore-mentioned, one central source being the Second Discourse), to construct a suggestion of this important message:^5^ These introductory paragraphs to *Morale Sensitive* provide a possible frame to understand the interrelations between Rousseau’s central concepts (such as *amour-propre*,* amour-de-soi*,* pitié*). Instead of representing mere separate “drives” or “feelings”, the interrelations between them form comprehensive “different manners-of-being” that completely change a person.

Along the lines of systems thinking, Rousseau depicts us as seamless parts in the net-of-coexistence, and was deeply unsatisfied with the consequences of people not realizing—or, feeling—the deep connectedness of all living. (Rousseau, 1997, p. 462, 1979, p. 275, 2005, p. 2.) In trying to understand ourselves, the first step is to be humble about how we view our species.^6^ We are not on top and separated from nature, but interdependent parts of it: All changes in the “*continuous flux”* (Rousseau, 1997, p. 418) of life are connected to one’s environment. Therefore, to really understand a living being entails taking into account its surroundings. Often, this deep relatedness that describes his thinking is referred to only in a human context (e.g., Dent, 1988, p. 130). I suggest it extends to the whole environment:

“*Everything is connected*” […] “*to know oneself*,* it is necessary to know man. In order to know man*,* it is necessary to study men; and to know men*,* it is necessary to study them in various times*,* in various places*”; we need to know where they live with the *“trees*,* plants*,* animals*,* mountains and valleys*,* rocks and seas*” (Rousseau 2007b, pp. 134–135).

In *Morale Sensitive*, he would have further addressed how *“Climate*,* seasons*,* sounds*,* colors*,* light*,* darkness*,* the elements*,* ailments*,* noise*,* silence*,* motion*,* rest […]*,* all this affect us in a profound way: we are embedded seamlessly in our environment”* (Rousseau, 2012, pp. 474–475).^7^ Thus, according to Rousseau, our ideas, sentiments, and actions are embedded and constantly modified by the environment and also by our “anterior impressions” regarding it:

*By examining within myself*,* and searching in others what could be the cause of* *these different manners of being*, *I discovered that*,* in a great measure they depended on the anterior impressions of external objects; and that*,* continually modified by our senses and organs*,* we*,* without knowing it*,* bore in our ideas*,* sentiments*,* and even actions*,* the effect of these modifications.* (Rousseau, 2012, pp. 474–475.)

In *Morale Sensitive*, Rousseau planned to solve how to avoid the contradictory situations of everyday life, where selfish gains clash with the common good. Winning those inside battles, one by one, he called virtuous (Rousseau, 1979, p. 444), but now, the aim was not in winning, but in *avoiding* them altogether: To change our manner-of-being so that these interests would not be on a collision course (“*true* virtue”), a state where *excessively* individualistic motives would not even arise. Rousseau believed we were not born with these inner contradictions, but end up with them through unsuccessful socialization processes (Rousseau, 1979, p. 41). In *Emilé*, Rousseau writes:

*Forced to divide ourselves between these different impulses*,* we follow a composite impulse which leads us to neither one goal nor the other. Thus*,* in conflict and floating during the whole course of our life*,* we end it without having been able to put ourselves in harmony with ourselves and without having been good either for ourselves or for others*. (Rousseau, 1979, p. 41.)

Here, a crucial distinction is made between “a composite impulse” and “harmony with ourselves”. The dual relation with the world never leaves us. This is true also with other organisms, but Rousseau suggested that with humans this duality is exceptionally intense: Excessive individualistic socialization may lead to a mental separation of ourselves from and above nature instead of harmoniously unifying the two impulses of life-sustenance both at the individual level, and at the level of the whole we are part of. Once separated, the attempt to create a composition of these now contradictory goals cannot but succeed poorly, ending up with no “truly" good for oneself or others. So, instead of having a continuous inner struggle, why not change the manner-of-being into one where the motives are not contradictory but complementary? To do that, Rousseau suggested they should be traced back to their *source*:

*It is undoubtedly more painful to an honest man to resist desires already formed*,* and which it is his duty to subdue*,* than to prevent*,* change*,* or modify the same desires in their source*,* were he capable of tracing them to it.* (Rousseau, 2012, pp. 474–475.)

What is this *source*, he does not specify in the short introduction to *Morale Sensitive.* Jean Starobinski (1988, p. 192) noted that “*to trace the hidden determinants of the present back to their source*” was a method Rousseau often used. Here, however, the reader has to do the tracing by referring to other writings.

I suggest that the balance between what the life-sustenance hypothesis calls self-sustenance and life-sustenance is the *source*: It is where the good for me becomes the good of the whole, not either-or. Thus, the different manners-of-being represent (1) an excessively individualistic, imbalanced manner-of-being, or (2) a balanced manner-of-being, where the *source* has been found, and these orientations in which we attend to the world are not contradictory but complementary. I suggest, further, that what Rousseau is after with these manners-of-being can be visualized as a *continuum*. This is because, as stated, the dual relation with the world never leaves us—we are both individuals and parts of wholes—and keeping the orientation to the world a balanced one is a *continuous*,* embedded*,* situation-sensitive process*, as the above quotations concerning *Morale Sensitive* suggest. It is in this process that Rousseau saw a continuous, socially-induced bias towards the excessive individualistic manner-of-being. In a perfect world (probably non-existent), the *source* would be permanently found, and we would be able to keep our manner-of-being in the world constantly in a state where the goals are complementary and unified. In reality, especially with humans as a highly social and cognitive species, life continuously fluctuates in between. Sometimes excessive individualism dominates, sometimes we are able to overcome it.

The idea to describe the manners-of-being in a continuum arose from realizing that for Rousseau, matters cannot be described in terms of stark polarities (as also discussed by Dent, 1992, p. 84). In *Emilé*, Rousseau wrote: *“it is impossible […] to give the same meanings to the same words. There is no language rich enough to furnish as many terms*,* turns*,* and phrases as our ideas can have modifications”* (Rousseau, 1979, p. 108). Despite this difficulty, he firmly believed that with sustained effort, the reader would find a clear system with one main principle (Rousseau, 1996, p. 629; Rousseau et al., 1990, p. xxii). This ambiguity of meanings arises, I suggest, from the dual relation with the world: The fact that our entire way of attending to the world is, at the same time to different degrees, both individualistic and holistic affects also what is meant with words. As an abstracting tool, language is not able to catch this dynamic process. Dent has also drawn attention to Rousseau’s central themes having two meanings: Rousseau discusses “morality of times”—generally esteemed behavior, including norms—and on the other hand, “true morality”, meaning truly good standards (Dent, 1992, p. 64). True morality is closely related to what Rousseau calls ‘the innate voice of Nature’. Rousseau also distinguishes dogmatic religions from an inner “natural religion”, which remains the same through times, different religions, and traditions—something that can only be known by feeling and which cannot be externally taught (Rousseau, 1979, p. 288, pp. 296–297). Similar distinctions can be seen in how he speaks, for example, about happiness, love, or virtue. “True”, “pure”, and “natural” are prefixes that Rousseau often uses when making these distinctions, but he is not, unfortunately, always consistent, leaving room for very different interpretations that may even contradict one another. My suggestion is that most of his central concepts have this inherent duality—sometimes he discusses the other extreme, sometimes the other, neither necessarily actualized in reality. For instance, the societal and ‘natural’ states of humans may never have existed as such, but rather, might be better understood to represent extremes of a continuum. Reality is always somewhere in the middle, “both-and” to different degrees.

In the becoming sections, Rousseau’s central terms are presented in light of this interpretative emphasis outlined above. The attempt is to build a coherent, continuum-based view of human existence that perhaps will not be able to solve all the contradictions in Rousseau’s philosophy, but might possibly provide a nuanced explanation for human-nature connection.

## Moral order, conscience, and the voice of nature

One central notion of Rousseau is “moral order”, possible to be interpreted in terms of self-centered ‘recognition’ from others (like Dent, 1992, pp. 162–163). Based on the ambiguity of words, I suggest there is another, ‘true’, or ‘natural’, form of it as well. Rousseau writes:

*There is some moral order wherever there is sentiment and intelligence. The difference is that the good man orders himself in relation to the whole*,* and the wicked one orders the whole in relation to himself. The latter makes himself the center of all things; the former measures his radius and keeps the circumference.* (Rousseau, 1979, pp. 291–292.)

In this sense, I interpret moral order to refer to the balanced manner-of-being. The idea is to transcend the individualistically rooted separation between me and the whole: Not, however, to forget one’s own needs and totally concentrate on the needs of others, for “I” am part of the whole as well. Rather, in line with systems thinking, to start from the whole and then acknowledge the parts—not to start from the parts (especially, *me*).^8^

Closely related to moral order is Rousseau’s notion of conscience. Dent (1992, p. 60) asserts that Rousseau never gave a full, systematic account of the functions of conscience. Interpretations differ.^9^ Mine comes close to Dent’s (1988, p. 229), who emphasizes that conscience is closely connected to the innate voice of Nature: Conscience is an expression, or awareness, of the demands of an inner order that constitutes our *true* needs and good (Dent, 1992, p. 60). Crucially, these true needs and our own good *include* the needs and good of others around us. Rousseau articulated one principle, ‘the only maxim to follow’: “*do your (own) good with as little harm to others as possible*”.^10^ Throughout this paper, I will try to point out that the good that Rousseau alludes to pertains not solely to individual good, for the “true” own good is only achieved by responding also to the needs of others.

The difference between moral order and conscience is very subtle. I interpret moral order to refer to the “ideal state” of which we have an innate sense of and conscience as the momentary experience, the concrete feelings that give us information of the distance from the ideal state at a given time—representing the fluctuation in the continuum of dual relation with the world. Compare this to sensing excessively rapid heartbeat and thus instinctively slowing down toward the ideal (unless there is a circumstantial reason for it being in overdrive). Conscience, in a similar manner, takes note of the circumstances and guides our actions accordingly, toward the situation-sensitive ideal state (moral order). Rousseau writes:

*For us*,* to exist is to feel; and our sensitivity is incontestably prior to our reason itself. Whatever the cause of our existence might be*,* it has provided for our preservation by giving us feelings in conformity to our nature; and one could not deny that at least those are innate. With regard to the individual these feelings are love of oneself*,* fear of pain and of death*,* and the desire for well-being. But if*,* as one cannot doubt*,* man is a sociable animal by his nature*,* or at least made to become so*,* he can be so only by means of other innate feelings relative to his species*. *And* *it is from the moral system formed by this double relation to oneself and one’s fellows that is born the natural impulse of the conscience*. (Rousseau 2007d, p. 196.)

Although in this context, Rousseau speaks of relations with our species, elsewhere he extends the same to other sensible beings (Rousseau, 1979, p. 225). Conscience is a natural and divine ‘impulse’ that arises from the double relation with the world (Cassirer, 1954, p. 109), and *“we feel it*,* like any other*,* as a form of pain or pleasure”* (Shklar, 1985, p. 38). It provides signals of an imbalanced relation in terms of life-sustenance, similar to how hunger signals an imbalance inside the self-sustaining organism.

### Amour-de-soi

Rousseau describes two innate “drives” or natural passions, which he calls *amour-de-soi* (love-of-self) and *pitié* (compassion). They are *“the instruments of our freedom: they tend to preserve us”* (Rousseau, 1979, p. 212). *Amour-de-soi* is the innate, primitive drive for self-preservation: *“Since each man is specially entrusted with his own preservation*,* the first and most important of his cares is and ought to be to watch over it constantly”* (Rousseau, 1979, p. 213)—thus, *amour-de-soi* represents self-sustenance, manifested species-specifically in organisms adjusting to their environments.

*Amour-de-soi* makes us strive for what is needed to survive, without harming other living beings unless there is a self-preservational need to do so: It is *“always good and always in conformity with order”* (Rousseau, 1979, pp. 212–213). If *order* in this context refers to true moral order, then this suggests a continuous balancing process between my needs and the needs of others with respect to the situation at hand. Furthermore, *Amour-de-soi* is *“the source of our passions*,* the origin and the principle of all the others*,* the only one born with man and which never leaves him so long as he lives. […] a primitive*,* innate passion*,* which is anterior to every other*,* and of which all others are in a sense only modifications”.* Thus, also *pitié* is a modification of *amour-de-soi*.

Some scholars suggest that *amour-de-soi* alludes to more than just survival needs. For example, Dent (1992, p. 31) asserts that a further aspect of *amour-de-soi* is a sense of competence as a successful agent in one’s own affairs. In my interpretation, this can be understood as a tool for self-survival: Imagine you had no sense of being successful or competent in your affairs. This kind of sense of self might lead to quitting the whole survival ordeal—why bother?

### Amour-propre

Rousseau introduced two concepts of self-love. *Amour-propre* is not natural like *amour-de-soi*, but a modification of it, arising along with social circles expanding.^11^ When not balanced by *pitié*, it becomes excessive and problematic, especially in circumstances of competition and comparison: The need to be valued and recognized evolves, leading to vanity, contempt, and envy, to the destruction of real happiness, to deliberate harming and revenge, power over others, excessive wealth of some, and cruelties such as slavery. (Rousseau, 2010, pp. 41–71.)

The exact moment Rousseau thought *amour-propre* appeared is debated. I see it as unresolvable because they work in an ever-fluctuating continuum, not in either-or form. As Rousseau maintains, *amour-de-soi* never leaves us—thus, *amour-propre*, as its modification, takes control only in some measure. Relevant are the dynamics between them.

*Amour-propre* is not the only way self-sustenance can become excessive. While in a normal situation, *amour-de-soi* is always in conformity with order, in harsh conditions such as continuous starvation, a deficiency in the ability to form a balanced manner-of-being may occur: “*There is a degree of degradation which takes away life from the soul*,* and the inner voice cannot make itself heard to someone who thinks only of feeding himself*” (Rousseau, 1979, p. 264). *Amour-propre*, however, goes further, striving to gain not just self-preservation but excessive resources, luxuries, and wealth, thus describing the problems facing the hyper-sociable and highly cognitive human-species, not all living beings in harsh conditions.

For a long time, *amour-propre* was taken as merely a negative drive, but during recent decades, attention has been drawn to its possible benign, positive forms (Dent, 1988; Neuhouser, 2008). In the continuum-approach of my interpretation, the earlier view of merely negative *amour-propre* could be envisioned as the excessive, unhealthy self-sustaining end of the continuum. The current view of *amour-propre*, on the other hand, would require that *amour-propre* is characteristically different from *amour-de-soi*, not straightforwardly its excessive form. For example, Neuhouser (2008) describes *amour-propre* distinctly as an excessive *need for recognition* (but see Kolodny [2010, p. 169] concerning the narrowness of this view). According to Dent (1988, p. 144), benign *amour-propre* takes also other people’s *need for recognition* as one’s own concern, thus striving for it just as much as one’s own. Thus, to put this in life-sustenance-terms, a benign *amour-propre* could contribute to the survival of the whole. Following the lines of double meanings of the words, the benign, healthy *amour-propre* would be a life-sustaining form of the need for recognition extended to others, and only excessive, self-centered need for recognition would be the troublemaker. This would explain why Rousseau (1979, p. 252) says *“Let us extend amour-propre to other beings. We shall transform it into a virtue”*: Virtue, not necessarily “true virtue”.

Related to the life-sustenance hypothesis, the emphasis when definin*g amour-propre* is not so much on the need for recognition, but more on the person’s *own* feeling of superiority, which justifies why “I” am entitled to more resources and good than others around me. While excessive need for recognition is certainly one means by which excessive *amour-propre* operates, the definition of Shklar (1985, p. 35) of *amour-propre* as the *desire for inequality* is in this context more apt. Excessive *amour-propre* aspires to attain more than mere survival needs—it drives toward one’s own advantage at the expense of others. Excessive *amour-propre* describes one, distinctly human, way in which dominating self-sustenance can become unhealthy. The benign form, which can be transformed into virtue, could—analogous to Dent’s description—be described as recognizing that others have similar, selfish *desires for inequality*, and striving to attain a situation where these are all taken into consideration together. Distinguishing between ‘virtue’ and ‘true virtue’ is very insightful in this context: While taming *amour-propre* to its benign forms approaches virtue by taking into account the needs of others and mine *one battle at a time*, it is not the same thing as finding the *source*, where this *need for inequality disappears*. In my understanding, Rousseau thought both of these routes are useful, but his careful analysis leads to the acknowledgement that they are, however, *distinct* from one another. At times, he proceeds by focusing on strengthening virtues induced by benign *amour-propre*, at other times, he considers how to pursue *true* virtuousness: That is, the *source*, the balanced manner-of-being, where *amour-propre*, defined as *excessive* selfishness, vanishes. Due to our societal arrangements, Rousseau did not think that true virtuousness could be something we could permanently achieve: This is why he also develops the other, virtuous, route.

Niko Kolodny (2010, p. 197) has expressed a worry that the contrast between an inflamed and a healthy *amour-propre* is not drawn sharply enough. The both-and-perspective does not demand such sharpness: Healthy and unhealthy *amour-propre* also function in a continuum. According to Dent (1988, pp. 62–63), a person with an excessive *amour-propre* “*destroys the possibility of any life-sustaining source in life for himself other than that of doing down others”.* Dent doubts, however, whether doing down others can ever be life-sustaining, for positional good is self-destroying in the long run. Thus, when *amour-de-soi* is modified to an excessive *amour-propre*, it ends up working against its own principle (Dent, 1988, p. 79): Not only against life-sustenance, but also against self-sustenance. From this point of view, it becomes even more urgent to tame *amour-propre* to its benign forms, given the acknowledgement that we cannot get rid of it permanently.

### Pitié

In the opposite scenario, *amour-de-soi* enlarges to *pitié*, a care for the well-being of other living beings around us like our own. Rousseau describes *pitié*, like the other concepts, in a two-fold manner (for another kind of, but as coherent a perspective than the one represented here, see Marks, 2006. In my interpretation, these views need not be incompatible). *Pitié* is a natural sentiment which *“hurries us without reflection to the assistance of those we see in distress”* and “*by moderating in every individual the activity of self-love*,* contributes to the mutual preservation of the whole species.”* (Rousseau, 2010, pp. 33–34.) Elsewhere, Rousseau (1979, p. 225) emphasizes that *pitié* should be felt for all beings, but it arises more easily towards members of our own species. Similarly to self-preservative feelings such as hunger, the distress *pitié* induces is alleviated when we respond accordingly: “*What would gratify us more than that of the happiness of someone else*,* an act of beneficence.*” (Rousseau 2007d, p. 194). Dent (1988, pp. 129–130), for instance, emphasizes the directness and immediacy of Rousseau’s *pitié*, and Shklar (1985, p. 46) denotes it as an instinctive reaction. A corresponding directness is attached to the “sympathetic instincts” that Darwin (1871, pp. 71, 77, 81–87) described, and contemporarily, to Frans De Waal’s (2008) altruistic impulses.

According to Rousseau, due to excessive *amour-propre*, *pitié* often gets socialized to the minimum, but, even *“the most corrupt souls cannot lose this first inclination completely […] there is no ferocious assassin who does not support a man falling in faint”* (Rousseau 2007d, p. 194; see also 1979, p. 288). Elsewhere, he continues:

*Finally we have a secure guide in this labyrinth of human errors*,* but it is not enough that it exists*,* it is necessary to know how to get to know it and to follow it. […] If it speaks to all hearts*,* […] why are there so few of them who hear it? Alas*,* it speaks to us in nature’s language*,* which everything has made us forget.* (Rousseau 2007d, p. 198.)

In the process of life surviving, both *amour-de-soi* and *pitié* are needed. So closely tied to each other they are, that *amour-de-soi* cannot even be ‘fulfilled’ without *pitié*:

*In whatever state a soul might be*,* there remains a feeling of pleasure in doing good that is never erased and that serves as a first foothold of all the other virtues*,* it is by this feeling cultivated that one succeeds in loving oneself*. (Rousseau 2007d, p. 201; See also Rousseau, 1997, p. 323.)

Thus, *pitié* also contributes to self-preservation—*amour-de-soi* “malfunctions” if harm to other life is caused without a self-preservational need. This is understandable from a systems biological point of view: Harming the whole we are part of sort of backfires. The importance of the *source*, where the *desires change* from excessively selfish toward complementary, balanced ones, becomes apparent: Ideally, it would prevent the malfunction. Dent (1988, p. 237) notices something similar when he says that *“Rousseau shows*,* in short*,* that we benefit from being good*,* and suffer in doing wrong or evil. […] we harm ourselves by yielding to such* [evil and unnatural] *feelings.”* Dent (1992, p. 31) also suggests that, contrary to the common assumption, *amour-de-soi* does not concern the good of persons “*wholly isolated from each other*”.

The life-sustenance hypothesis assumes that *pitié* is experienced by all life forms. Rousseau does not explicitly go this far, but he does not restrict it to only humans either. *Pitié* is

…*so natural*,* that the beasts themselves sometimes give evident signs of it […] with what reluctance are horses known to trample upon living bodies; one animal never passes unmoved by the dead carcass of another animal of the same species* (Rousseau, 2010, p. 32).

In practice, as *pitié* is expressed species-specifically, in less social and cooperative species than human beings, *pitié* might show more clearly in not harming other life needlessly than in actual helping (although there are also numerous examples of helping, see e.g., De Waal, 1996). As a hyper-social species, humans actively pursue the well-being of others. Although studies suggest that empathetic responses are phylogenetically ancient, and thus shared by birds and mammals (De Waal, 2008), the behavioral effects vary between species. I suggest that *pitié* is a more comprehensive and nuanced concept than mere empathy: It involves the species-specific, situation-sensitive behavioral manifestations. The “doing good” that induces a feeling of pleasure that, in turn, contributes to the fulfilment of *amour-de-soi* is not the same throughout situations, individuals, or species.

Other interpretations of *pitié* are, of course, possible. The interpretation of *pitié* as a mere empathetic physical response would lead to different conclusions compared to the ones in this paper. On the other hand, the difference could also be only conceptual: While referring with “*pitié”* strictly to empathetic response, the wider action-feedback-loop could still be acknowledged as existing in Rousseau’s thinking, with a different conceptual characterisation.

Although we have a natural propensity toward *pitié*, the environment has to support its development. Socialization can also hinder it and modify *amour-de-soi* toward excessive *amour-propre* (Rousseau, 1979, pp. 235, 252). In reality, both happen: The result comes down to the dynamics. Education plays an important role: *“The best education […] tries to establish a harmony between the self and the environment”* (Shklar, 1985, p. 159). I have addressed the educational thinking of Rousseau in more detail elsewhere (Salmi, 2025b).

In Dent’s translation (1988, p. 97), *pitié* induces “*a natural repugnance at seeing any other sensible being*,* and particularly any of our own species suffer pain or death”. Pitié* is, thus, directed to any sensible beings. It extends beyond our human-in-groups and species, even to the Universe itself:

*The Universe is a large family of which we are all members; thus we are also obliged to be acquainted with its situation and its interests: however small the extent of a private individual’s power might be*,* he is always in a condition to make himself useful somewhere to the great body of which he makes up a part.* (Rousseau, 2005, p. 2.)

According to Rousseau (1979, p. 225, 1998, p. 307), *pitié* is easier to invoke toward organisms similar or close to us, although we *should* protect all life. This stems from the difficulties of imagining the inner life of a being very different or distant; “*This*,* I think*,* is one of the causes which hardens us more to the ills of animals than to those of men*,* although* *the common sensibility ought to make us identify with them equally”* (Rousseau, 1979, p. 225, emphasis added). Therefore, Neuhouser (2008, pp. 222–223) has suggested that *pitié* is unreliable because it naturally favors in-group and similar beings, and even if it did not, it is “blind”—only with reason can we decide who “deserves” helping. Rousseau did emphasize that *pitié* needs to be “enlightened by reason”—and reasonably so, since we are a highly cognitive species. However, he also stressed it is educationally important to *prevent* the selectiveness of *pitié* (Rousseau, 1979, pp. 252–253)—perhaps because reason will end up in a different solution depending on the balance between the manners-of-beings. Although in situations of choice of whom to help, we usually do favor our kin and in-group, the difference comes to light when there is no choice to be made except whether to help or not. While *amour-propre* would ask “Why help, what’s in it for me?”, *pitié* would ask “Why not help?”, with only self-preservative needs overriding its force.^12^

Finally, a few words about the interplay between *pitié* and conscience. Shklar (1985, p. 61) states that conscience is an instinct of self-love extended to others through identification. While *pitié* is only concerned with the well-being of others and, without *amour-de-soi* balancing it, would help others even self-destructively, conscience takes into account both *amour-de-soi* and *pitié* and balances between them in situ: It arises from the double relation with the world. Furthermore, Dent (1992, p. 61) has pointed out that *“In the case of conscience*,* it is the requirements of our whole nature as an element of God’s design that is becoming manifest*,* whereas in the case of compassion it is only one part of the whole”*. Thus, *pitié* has a sharper radar: It helps the ones in distress one at a time, while conscience would have a larger picture of the whole in mind, relating to the situation and interests of the whole family of the universe at once (Rousseau, 2005, p. 2), me included.

## Perfectibility, free will, and the role of reason

Rousseau scholars (e.g. Neuhouser, 2012, p. 374) have drawn attention to Rousseau’s contention that human nature consists of *four* basic elements: Along with *amour-de-soi* and *pitié*, also perfectibility and free will. Neuhouser describes perfectibility as *“a host of latent cognitive capacities—for language*,* thought*,* imagination*,* and so on”*, and free will as “*the ability to follow or resist—to act on or say no to—what could loosely be called ‘instinct*,*’ or the promptings of nature*”. Relating to perfectibility, Kolodny (2010, p. 186) even suggests that “*amour de soi encompasses a drive*,* not necessarily self-conscious*,* for self-perfection”*, concluding thus that *amour-de-soi* would have not only a self-preservational side but also a “progressive” side (Kolodny, 2010, p. 167). Dent describes perfectibility as plasticity, flexibility, and adaptability. In combination, perfectibility could be described as the distinctly human flexibility in adapting to vastly different environments by using progressive enhancement of cognitive and other capacities. The other side of the coin is, in Gauthier’s (2016, p. 26) words, that: “*Only a perfectible being can become degraded*”—this useful flexibility can also turn against itself, by creating continuously new desires and passions to fulfil (Gauthier 2006, p. 8). Here again, I think the most useful way to approach the matter is to picture it in a continuously fluctuating continuum.

Dent (1988, p. 97, 1992, pp. 189–191) takes perfectibility and free will as components of *amour-de-soi.* While *amour-de-soi* and *pitié* are something that all species share, perfectibility and free will are not in the same manner fundamental drives that ensure the sustenance of life, but rather, I suggest, human-specific adaptations to implement them (not to say that other species could not acquire something similar), means by which our species has survived and brought to practice our dual relation with the world.

The role of high cognitive abilities—i.e., reason—for Rousseau is highly debated. A view compatible with my approach would be that while conscience is our compass, directing us toward the destination, reason is like a map, a tool of abstraction to help us reach it. A map is useless without knowing where you stand in relation to your surroundings. Rousseau writes: *“it is not true that the precepts of natural law are founded on reason alone. They have a base more solid and sure. Love of men derived from love of self is the principle of human justice”* (Rousseau, 1979, p. 235). Moreover, *“the human species would long ago have ceased to exist*,* had it depended entirely for its preservation on the reasonings of the individuals that compose it”* (Rousseau, 2010, p. 34).^13^ On the other hand, it must be remembered that Rousseau saw the development of our species as irreversible: Thus, some amount of reasoning will always be involved.

## The contagiousness of the drives

A topic that has received little attention, but for the life-sustenance hypothesis is central, is that excessive *amour-propre* exhibits a contagious nature; *“only the continual practice of beneficence can protect the best hearts from the contagion of the ambitious”* (Rousseau, 1997, p. 249). Ambition is caused by *amour-propre* with its competitiveness. Moreover, it is an extremely difficult task for “*the upright man to preserve his virtue ever pure while keeping constant company with the wicked*” (Rousseau, 1997, p. 436)*—*wicked referring to the excessively *amour-proprean* individuals, the opposite of the *purely* virtuous. Furthermore, to find the ‘voice of Nature’, in *Moral Letters* Rousseau advises to avoid urban and social life by retreating to nature and solitude: It helps to minimize the amount of contagious, excessive *amour-propre* around. Later, when the inner balance between the orientations has strengthened, one can keep it up also in social circumstances with only short retrieves when needed. (Rousseau 2007d, pp. 198–200, 1997, p. 249.) There is some indication that Rousseau also took *pitié* to be contagious: “*By associating with these happy spouses […] my heart is gradually coming into unison with theirs*,* just as one’s voice takes on unwittingly the tone of those with whom one is conversing”* (Rousseau, 1997, p. 432). “Happiness” parallels the balanced manners-of-beings (see next section).

One explanation for the contagiousness could be, following Dent (1988, p. 64), that excessive *amour-propre* is institutionalized. Another might be physiological synchronicity: During social interactions, our autonomic nervous system responses are automatically attuned to the states of others. Thus, according to the life-sustenance hypothesis, the biomarkers of the orientations, such as cortisol levels, heartbeats, brainwaves, and breathing—all markers of both (1) self-preservative stress responding and (2) nature connectedness*—*are aligned with the levels of others around us, even in the absence of any verbal or nonverbal cues. This happens especially with loved ones, but has been detected between strangers and even between different species. As a highly social species, humans seem specifically sensitive to this contagiousness. (Herrando 2021; Sundman et al., 2019; Salmi, 2025a.)^14^

## Harmony and true happiness: nature connectedness in Rousseau

Now we are prepared for what Rousseau had to say about the research topics of human-nature connection studies. Ernst Cassirer (1954, pp. 49–50) has insightfully suggested that Rousseau’s “return to nature” was meant to distinguish between what man is and what he has made of himself, and that only by *genuine self-examination* can one approach this distinction. Indeed, in the ‘fourth solution’ to our contemporary human condition, Rousseau turns inwards to find inner peace instead of political or educational solutions. Whether Rousseau-scholars see this path as a successful one varies, but in the context of nature connectedness research and theoretical frameworks explaining it, it is the central one.

In line with contemporary research, Rousseau acknowledges nature connectedness as both a temporary state of mind and a more stable feature. He calls the temporary state ‘being in *harmony* with nature’: A nonverbal, noncognitive, embodied experience of a fleeting moment of pure feeling of existence where the limits of subject and object melt away—a timeless state, where one feels or sees nothing particular, but still everything: The only thing one senses is existence itself. It feels like getting immersed in the whole of nature; as if nature would fill all the senses; one’s existence becomes the existence of the whole of nature. (Rousseau, 1927, pp. 112–114, 138–143; 2007c, 165–168.)^15^ In the life-sustenance hypothesis, harmony is the extreme end of the continuum of the dual relation with the world, where the sense of self vanishes completely, and one only feels unity with the whole.

Harmony is a relatively rare encounter that cannot be sustained continuously: Self-preservation requires self-focus. However, experiencing harmony may stimulate a more permanent change, where the dominance of excessive *amour-proprean* manner-of-being may shift toward a balanced one: The *source* mentioned in *Morale Sensitive*. I suggest that what Rousseau calls *true happiness* equals this more stable, permanent state. Rousseau writes: “*true happiness cannot be described; it can only be felt*,* and felt the more*,* the less it can be described*,* since it is not the result of a number of facts*,* but is a permanent condition.”* (Rousseau, 1996, p. 229, see also 2007d, p. 181).

According to Dent, Rousseau never systematically defined happiness, and in his later years, he enjoyed the *“trance-like absorption in nature”*: Instead of happiness, settling for contentment, peace of mind, and tranquillity. Dent points out that the distinction Rousseau is trying to draw here is not entirely clear. (Dent, 1992, pp. 132–133.) In my interpretation, following the ambiguity of words, this tranquillity equals *true* happiness, as distinguished from shallow *amour-proprean* happiness.

What makes it particularly accurate to parallel harmony and true happiness with nature connectedness is that they are more easily achieved when in nature instead of cities full of people and built environments: In the beginning, it is not necessary to learn *“how to be alone in the middle of the social world”* (Rousseau 2007d, p. 199), but when the manners-of-beings are balanced, a return to societal circumstances is possible:

[…] *Remove the objects that must distract you until their presence no longer distracts you. Then live ceaselessly in their midst*,* you will know very well when it will be necessary for you to find yourself with yourself again. Thus I am not at all saying to you: leave society.* (Rousseau 2007d, p. 199; See also Rousseau, 1997, p. 64.)

Solitude in itself is ineffective: “*Alone in the city between four walls resembles a prison*” (Rousseau 2007d, pp. 199–200). It is more difficult to feel embodied connectedness with life when detached from it.^16^ Distractions may also originate from one’s own thoughts; harmony shares some common ground with meditative practices (Rousseau 2007d, p. 200), whose beneficial effects and link with nature connectedness are well established (e.g., Schutte & Malouf, 2018).

As the balance in the *source*, true happiness can only be achieved by forgetting one’s own excess advantages. Hence, one possible way toward it is activating *pitié*: i.e., to strengthen the other end of the continuum. As Rousseau puts it, *“It is in doing good that we become good*” (Rousseau, 1979, p. 250; see also 2007d, p. 194 and p. 203). This might suggest that by acting kindly, one can influence the dynamics between the manners-of-being and, thus, one’s true happiness.

Rousseau (2007a, p. 50) notes that “*Happiness is too constant a condition and man too mutable a being for the one suit the other”*. We are not able to maintain a constant equilibrium with regard to the life-sustenance continuum. Yet, nor should we: Life is by definition a homeostatic process, where a constant equilibrium is impossible (Damasio, 2018, pp. 40–49) and some fluctuation is inevitable: Stasis, in fact, means death. Constancy should not even be a goal. However, it matters *where* we fluctuate in terms of “baselines” (ranges of typical fluctuation): If our baseline is dangerously too close to the extreme ends of any of the homeostatic life regulations, it means trouble. So too here.

## Discussion

In the past pages, I have sketched a systems approach to the life work of Jean-Jacques Rousseau. Outlining it thoroughly is a task impossible in just one paper. One shortcoming relates to his political writings, and the common interpretation of Rousseau’s inherent ambiguousness, emphasizing the importance of nature, but then giving anthropocentric guidance to modify nature for the good of people in societies. However, if one starts with his moral psychology, then it becomes apparent that the good of “me” and the people *is* the good of nature: Thus, on some occasions, by providing circumstances where people have opportunities to get immersed in nature, Rousseau might want to steer the manner-of-being in the world towards life-sustenance.^17^ What may seem on the surface to be anthropocentric, or even selfish (in the domestic solutions), could be for the good of all. On the other hand, Rousseau’s political writings might mirror a solution given up on hope for stability with regard to the inner balance between the orientations, and offer institutionalized restrictions to replace it in an inherently *amour-proprean* human-world (transforming *amour-propre* to its benign forms, but giving up on true virtue). Like I have tried to argue, he may have advanced both routes at the same time: Strengthening pitié and true happiness, but also transforming *amour-propre* to its benign forms.

Reconciling in detail the systems approach with Rousseau’s political thought is out of the scope of this paper. Perhaps it cannot be done, and Rousseau remains controversial. However, in the current times of polycrisis, I think more important than whether the systems approach to Rousseau is coherent or not, is that through this interpretation, it is possible to provide coherence to the phenomenon contemporarily studied under human-nature connection research. Harmony equals nature connectedness as an immersive, temporary state of mind, and true happiness equals its more permanent form, the *source*. Nature connectedness as a multidimensional phenomenon is explained with life-sustenance manifesting in the entire way of being, covering all dimensions from emotional to cognitive ones. Accordingly, although Rousseau pays considerable attention to the instinctive origin of *pitié*, we are still a highly cognitive species: Therefore, how we as a species manifest life-sustenance is, contemporarily, often in the lines of social virtues induced by benign *amour-propre* and rational deliberations. This is echoed in the human-nature studies: Whether it is the emotional “love of life”, cognitive recognition of nature’s intrinsic value, or measurements regarding experiences of the state-like connectedness (harmony), they all correlate—people who score high in one, tend to score high in others as well, representing a more comprehensive manner-of-being, rather than just one distinct feature (i.e., the biophilia hypothesis’ adaptive “affiliation of life and life-like processes”) that in our species would have been selected for self-preservative ends. Life-sustenance brings in an alternative point of view: Perhaps the underlying basis for nature connectedness is not in self-preservative adaptations but in more basic, all-life-describing orientations of autonomy and integration described in systems biology, and anticipated by Rousseau with his dual relation with the world.

One key problem in current theoretical frameworks for nature connectedness is the relation between the physical and the psychological connection. An explanation based on Rousseau’s system of thought might be that trying to balance the orientations, the *source*, is best reached in nature (nature exposure), because there is (1) less contagious *amour-propre* around, and one is (2) more exposed to other life forms than one would be inside four walls. Rousseau also held that nature exposure in itself can heal (Rousseau, 1996, p. 226, 237, 448, 631, 2007, pp. 189–200, 310, 1979, p. 474; for a contemporary scientific approach, see Robinson & Breed, 2020).

Yet another problem of the current frameworks is whether intrinsic selfishness is hidden in Naess’ deep ecology: When we expand our narrow ego to an ecological Self, are we still functioning from self-centered standpoints, only with an extension on what we consider as the self. The same criticism applies to Rousseau, for *pitié* has an empathetic basis. In my interpretation, however, *pitié* and conscience are especially insightful terms when they are understood as signs to initiate action, informing the organism that there is an imbalance that needs a response. They function like *tentacles*, showing directions for responsiveness. Thus, while Rousseau (2007d, p. 179) suggests that happiness is the object of human life, in my view, true happiness should not be seen as a goal *in itself*, but as a *signal*: Similar to the feeling of being full instead of hungry signals that the energy levels are in balance, true happiness signals that the level of overall life-sustenance is currently in balance. When there is an imbalance, we feel discomfort, distress, or emptiness because something that could be described as a basic need is not fulfilled.^18^ Crucially, in a balanced manner-of-being, the needs based on self-sustenance and life-sustenance unite as one, and when fulfilled, the distress vanishes. Unlike in Naess’ theory, where nature becomes part of me, here, the needs are *aligned*. *Amour-propre* tries to fill this void of unmet needs with luxuries, wealth, and admiration, but never truly succeeds. Its methods are flawed: It is only in response to the needs of others that these basic needs can be fulfilled. This is, at its core, how Rousseau thinks that being good to other living beings *directly* affects our own well-being—hence, at least partly, this directness can (unlike Naess’ ecological Self, which concentrates on the proenvironmental effects) possibly explain some of the well-being benefits of nature connectedness (for more, see Salmi,  2025a).

Rousseau’s deep acknowledgment of the dual nature of all life is in the background of all his thinking. He saw the development of our species—the expanding sociality, ever-growing group sizes, the improvement of cognitive abilities—as largely inevitable in terms of survival. While the advantages of progressive improvement, especially during modernity, are substantial, it has also induced excessive individualism with its consequences on our well-being and how we treat life around us. In Rousseau’s words: *“If the abuses of this new condition did not often degrade him below that from which he has emerged*,* he ought to bless continually the wonderful moment that released him from it forever*.” (Rousseau, 2002, pp. 166–167.) The tension between the orientations—the continuum—has always been there, but with our species, currently, the ravine between the extremities has expanded because the *amour-proprean* desires lead further and further apart from a more holistic manner-of-being. The routes to fix the situation are either to focus on taming *amour-propre* to its benign forms, strengthen *pitié*, or, through experiences of harmony, slowly approach a more stable, trait-like nature connectedness.

Both self-sustenance and life-sustenance contribute to the survival of life. If life (the net-of-coexistence) were not equipped with (1) safeguarding the survival of its organisms and (2) the survival of the whole of them, it would not, arguably, have prevailed. After all, we are as dependent on the surrounding whole as we are on the parts that constitute us. Every organism then needs species-specific features that safeguard both of these intertwining survival aspects. The life-sustenance hypothesis posits that the experiences of nature connectedness and altruistic impulses are human-specific manifestations of the life-sustaining orientation. Comparable to the feeling of hunger manifesting the self-sustaining orientation, they directly influence our own well-being when responded to.

The constantly fluctuating process of manners-of-being depends not only on the particular situation we are in—danger, hunger, rest, social bonding, spotting someone in distress—but also on the baselines, developed while embedded in the environment. As a highly cognitive species, we then use reasoning to find out how to alleviate the direct needs that self-sustenance and life-sustenance bring forth: How to find food, to self-preserve without excessive harm to other life, or to help the ones in need. All this directly influences our own well-being. As *amour-de-soi* cannot be fulfilled without *pitié*, personal well-being cannot be fully realized without taking into account the well-being of life around.

In the life-sustenance hypothesis, I draw parallels with *amour-propre* and the excessive, prolonged stress responses of people in modern, individualistic cultures, caused by anticipatory, social-psychological stressors (Sapolsky, 2004; Salmi, 2025a). Homeostatic stress responses are used by all organisms, from the cellular level to us humans, for self-preservative functions. However, humans initiate stress responses also for social reasons, and in an anticipatory manner. Furthermore, research has shown that human-nature connection correlates with lowered stress levels (Shuda et al., 2020).

Some scholars have emphasized similar stress-related (in its original, self-preservative sense) aspects in Rousseau without mentioning stress responding per se. Dent (1992, p. 35), for example, writes that *amour-propre* is a desire to have what belongs to us as equal members of a group. Problems start when we feel threatened by others to deny us this equality, and *“It is in response to such a* *perceived threat* *that amour-propre takes on an excessive*,* deformed character*,* and in its own* *defense* *seeks to deprive others.”* Thus, *amour-propre* induces a flawed perception of threat to self and, consequently, a self-defensive response (i.e., a stress response). Shklar (1985, 38), in turn, mentions that *amour-propre* is a *“compulsive response to every pressure from the social environment*.” (Shklar, 1985, p. 88).

Spending time in nature is one way to reach a manner-of-being, in which taking into account the sustenance of other surrounding life is balanced with one’s own needs. A fundamental shift in our perspective with regard to our place within the entirety of life, from a supposed on-top-spot to one-among-the-soup, is necessary from both self- and life-sustenance perspectives: *‘it is in forgetting oneself that one works for oneself*” (Rousseau, 1979, p. 313). Due to the contagiousness of the drives, by finding one’s own inner balance, one also contributes to the inner balance of those around. Perhaps, if there are enough of these little streams of expanding balanced manners-of-being, vast rivers may eventually flow from them.

## Data Availability

Not applicable.

## Footnotes

1 The decision to proceed in this backward manner to first introduce the life-sustenance hypothesis is strictly to provide flow and readability to the paper; otherwise, it might have seemed quite random to bring up some non-central features of Rousseau’s thinking. Originally, I started out with a quest to find out whether my interpretation of Rousseau would get any contemporary scientific backup, and indeed, found that the research on human-nature connection gives empirical evidence to his concepts. I soon realized that Rousseau is potentially better at explaining the empirical results than the existing frameworks. The life-sustenance hypothesis that resulted is not a word-for-word Rousseau-replica, but a further development of it, as I will indicate in the text when appropriate. Therefore, the purpose of this project is not anachronistic: The hypothesis is an integration of Rousseau and contemporary thinking, not straightforwardly derivative from any individual source. With this paper, however, I want to pay my respects to the original thinker behind the further developments.

2 A historical link between Rousseau and systems biology may exist. The holistic view of life, inherent in systems biology, can be traced back to Immanuel Kant (Moreno & Mossio, 2015), who is known for appreciating Rousseau. Exploring this possible link is, however, out of the scope of this article.

3 Compare to Aldo Leopold: “*A thing is right when it tends to preserve the integrity*,* stability*,* and beauty of the biotic community. It is wrong when it tends otherwise”* (Leopold, 1949, p. 224).

4 I have also used translations in Rousseau, 1996, 397–398; Rosen, 2004, 17.

5 Also, Sonenscher (2020) and Rosen (2004) have emphasized *Morale Sensitive* from different angles.

6
*“Let us not say in our imbecile vanity that man is the King of the world*,* that the sun*,* the stars*,* the firmament*,* the air*,* the earth*,* the sea are made for him*,* that the plants germinate for his subsistence*,* that the animals live so that he might devour them”* (Rousseau 2007d, p. 189).

7 A similar worldview is shared later by Charles Darwin (2009, 204, 214): Adaptation always occurs in relation to the environment that an organism inhabits.

8 Compare this to Shklar’s (1985, 202) presentation of Rousseau’s ‘body politic’ as almost like an ecosystem: *“The body politic*,* like healthy man in nature*,* does not neglect its self-preservation. An integrated whole*,* it does no injury to itself*,* but on the contrary avoids any source of pain. When the health of this body is gone*,* then its instincts*,* the peope’s self-love*,* no longer operates effectively.”*

9 “For a different perspective, see, for instance, Marks (2006). These different aspects need not be exclusive: Conscience can be considered as one of the concepts that has inherently different meanings.

10 “Fais ton bien avec le moindre mal d’autrui qu’il est possible” (Rousseau, 2015, p. 40).

11 Rousseau describes *amour-propre* as “artificial”, not “natural”. This relates to the assumption prevalent in the Second Discourse, i.e., his famous “natural state” of humans, not yet social creatures but solitary, with *amour-propre* still non-existent. However, in the preface of the Second Discourse, Rousseau himself casts doubt whether or not the natural state ever existed in reality: Thus, the point of drawing this picture of a “pure” natural state was perhaps more a rhetorical one, to bring to light the extremities of the continuum, regardless of whether they never have or ever will exist as such. Thus, “artificial” can be interpreted not as something that “was not there to begin with”, but rather something that “originates in specific kinds of social relations”. In comparison to natural *pitié*, the difference is that, regardless of whether you are a member of a social species or not, *pitié* develops in the right kind of species-specific circumstances, while *amour-propre* develops only to highly social and cognitive species. Accordingly, ants, octopuses, and orangutans can be pictured to manifest *pitié*, but not *amour-propre.*

12 These are moral-psychological, not normative remarks—accordingly, the life-sustenance hypothesis is not a normative account that could give general guidelines to social dilemmas. It is fundamentally situation-sensitive.

13 Contrary interpretation of Cassirer (1954, 101–102) argues Rousseau *eliminating* feelings from ethics, and *pitié* not being able to take an active interest in others. More agreeable with my interpretation is Shklar (1985, 62 and 72), suggesting that conscience inspires feelings and action, and reason is needed to guide them.

14 My intention is not to suggest materialistic worldviews to Rousseau’s account. I endorse a view of the mind-body problem similar to naturalistic monism in Jylkkä and Railo (2019): the mental and the physical cannot be reduced to each other or be derived from each other, but are the same from different perspectives.

15 In forming a comprehensive understanding of harmony, I have also used Rousseau’s descriptions of an experience of revelation: e.g., He describes a moment when the experiencer is looking at the sky: “*Suddenly this so familiar sight*,* that usually touched him so little*,* left him now filled with amazement and adoration. […] a new universe opened up to his meditation*,* he sensed an invisible chain linking all beings together. The divinity of Nature was revealed to his understanding*” (Rousseau 2007c, 165–168). I deem this choice justified, for (1) his central concepts are often described in both divine and natural circumstances (i.e., innate, moral “voice of Nature” equals divine “voice of God”). Furthermore, Rousseau thought (2) all religions and traditions are based on the same, innate ‘voice’, so divinity is not limited to any particular religion. Finally, (3) contemporary research acknowledges the similarities between different immersive state of minds, referred combinedly as self-transcendent experiences.

16 Solitary walks in nature *“possessed a still greater charm*,* because the heart had greater freedom of expansion”* (Rousseau, 1996, p. 237). Compare this expansion to that of *amour-de-soi* expanding to *pitié*. (See also Rousseau, 1996, p. 100; 229; 448; 631).

17 The unification of my good to the good of the whole should also ease the worries of Rousseau expressing totalitarian ideas: They cannot be in contradiction. For more on reconciling contemporary research on psychological nature connectedness and moral psychology by the means of the life-sustenance hypothesis, see Janhonen et al., 2026. 

18 Hurly and Walker (2019) discuss nature connectedness as a basic need.
